# Prevalence and Clinical Significance of Symptoms at Ultra High Risk for Psychosis in Children and Adolescents with Obsessive–Compulsive Disorder: Is There an Association with Global, Role, and Social Functioning?

**DOI:** 10.3390/brainsci8100181

**Published:** 2018-09-30

**Authors:** Roberto Averna, Maria Pontillo, Francesco Demaria, Marco Armando, Ornella Santonastaso, Maria Laura Pucciarini, Maria Cristina Tata, Francesco Mancini, Stefano Vicari

**Affiliations:** 1 Child and Adolescence Neuropsychiatry Unit, Department of Neuroscience, Children Hospital Bambino Gesù, Piazza Sant’Onofrio 4, 00165 Rome, Italy; roberto.averna@opbg.net (R.A.); francesco.demaria@opbg.net (F.D.); ornella.santonastaso@opbg.net (O.S.); mlaura.pucciarini@opbg.net (M.L.P.); mariacristina.tata@opbg.net (M.C.T.); stefano.vicari@opbg.net (S.V.); 2 Office Médico-Pédagogique Research Unit, Department of Psychiatry, University of Geneva School of Medicine, 1211 Geneva, Switzerland; marco.armando@unige.ch; 3 Scuola di Psicoterapia Cognitiva APC-SPC, Viale Castro Pretorio, 116, 00185 Rome, Italy; mancini@apc.it

**Keywords:** obsessive–compulsive disorder, psychosis, ultra-high risk, functioning, child and adolescent psychiatry

## Abstract

In literature nothing is known about the clinical significance of Ultra High Risk (UHR) symptoms in children and adolescents with diagnosis of obsessive–compulsive disorder (OCD). In this study, we examined the prevalence of UHR symptoms and their relationship with severity of obsessive–compulsive symptomatology, global, social, and role functioning, and level of associated depressive symptoms in a clinical sample (*n* = 51) of children and adolescents aged between 8 and 17 years with a diagnosis of OCD. The prevalence of UHR symptoms in this sample was 43.1%. We divided the whole sample into two groups: children and adolescents with OCD and UHR symptoms (*n* = 22) and children and adolescents with OCD without UHR symptoms (*n* = 29). Our findings suggest that the group with OCD and UHR symptoms shows worse global, social, and role functioning than the group with OCD without UHR symptoms. No differences were found on the severity of obsessive–compulsive symptomatology, the number of psychiatric diagnoses associated, and the level of depressive symptoms. The presence of UHR symptoms in children and adolescents with OCD could cause significant functional impairment and should be considered in order to plan specific and targeted therapeutic interventions.

## 1. Introduction

Obsessive–compulsive disorder (OCD) is a neuropsychiatric disorder characterized by intrusive, repetitive, unwanted thoughts, and/or repetitive neutralizing behaviors or mental acts [1] that affects 1–2% of children and adolescents [2]. Indeed, in about 50% of adult OCD cases, individuals report that their symptoms started before the age of 18 years [3]. Obsessive–compulsive disorder (OCD) is associated with significant reduction of quality of life, similar in magnitude to patients with schizophrenia [3,4].

Nonetheless, OCD often goes unrecognized and thus untreated [5]. To formulate a correct diagnosis, it is important to detect OCD early and to recognize the overlap with symptoms of related psychiatric disorders

In this respect, it has been increasingly proposed that OCD may be in comorbidity with different types of psychotic disorders, including schizophrenia, schizoaffective disorder, and mood disorders with psychotic features [6]. In addition, both bipolar depression and unipolar depression may be found in comorbidity with OCD and share common symptomatic and functional impairments in children and adolescents. Various brain imaging techniques have been used to investigate the integrity of brain white and gray matter in these disorders. For example, Serafini et al. [7] showed that unipolar and bipolar disorders present both shared and distinctive impairments in the white and grey matter compartments with more white matter abnormalities detected in bipolar disease than in unipolar disease.

Previous studies that explored the relationship between OCD and psychosis have only been conducted on adults with chronic schizophrenia [8,9,10]. In these studies, OCD occurs in 7.8–31.7% of patients with schizophrenia. Some researchers [11] proposed that the development of OCD in schizophrenia may be associated with effects of atypical antipsychotic medications on serotonergic sites associated with basal ganglia functioning, suggesting that OCD may be the consequence of treatment in some individuals with schizophrenia. 

Thus it remains unclear whether OCD bears any relationship to the emergence of psychosis and schizophrenia, or whether the two disorders are independent. In order to assess this relationship, it would be interesting to study the clinical picture and the possible evolution of patients with initial diagnosis of OCD associated with prodromal symptoms of psychosis (Ultra high risk symptoms; UHR) and not frank psychotic symptoms [11]. Like this, it would be possible to study the clinical significance of UHR symptoms in patients where OCD appears early and where there is not a long history of untreated illness or pharmacological treatments.

To date, there has been only one study conducted on a sample of young people at ultra-high risk (UHR) for developing psychosis and OCD. In this, Niendam et al. [12] reported that UHR individuals with OCD exhibited greater levels of depression, suicidality, and positive and negative symptoms compared to individuals with UHR symptoms but without OCD diagnosis. However, baseline OCD in individuals UHR was associated with a statistical trend towards lower rates of conversion to psychosis over the follow-up period. Further, while self-reported obsessive–compulsive symptoms (OCS) remained temporally stable among UHR individuals who did not convert to psychosis, OCS declined among those UHR individuals who developed psychosis. However, this analysis relied on self-reported symptoms and it remains unclear whether persistence, remission, or de novo OCD over time are associated with changes that contribute to conversion to psychosis. In addition, in Niendam et al. [12] there were recruited participants of 12 years old or older, with a resulting lack of information on children from 8 to 11 years old.

In this study, we examined the prevalence of UHR symptoms and their relationship with severity of obsessive–compulsive symptomatology, global, social, and role functioning, and level of associated depressive symptoms in a sample of children and adolescents aged 8–17 years with OCD diagnosis. We hypothesize that children and adolescents with OCD and UHR symptoms could show a worse global, social and role functioning than children and adolescents with OCD but without UHR symptoms.

Therefore, the presence of UHR symptoms could exacerbate the clinical picture of children and adolescents with OCD diagnosis determining the need for early and targeted therapeutic interventions. 

Finally, to address the limitation of self-reported questionnaires, UHR symptoms and presence of OCD were assessed using semi-structured-interviews with levels of reliability and validity superior to those of self-report questionnaires (Children’s Yale–Brown Obsessive Compulsive Scale (CY-BOCS) [13], and Structured Interview for Psychosis-Risk Syndrome (SIPS/SOPS) [14] respectively).

## 2. Materials and Methods

### 2.1. Participants

Participants in this study were 51 (16 female, 35 male) children and adolescents aged 8–17 years (mean age: 13.4 ± 2.9 years) consecutively admitted to the Child and Adolescent Neuropsychiatry Unit of the Clinical and Research Hospital Bambino Gesù of Rome with an OCD diagnosis between 2016 and 2017. 

Inclusion criteria were presence of OCD as a primary diagnosis based on DSM-5 [1] and IQ ≥70. Exclusion criteria was a past or present psychosis. All participants were drug-naïve patients at the time of clinical assessment. The study was approved by the Ethics Committee of the Children’s Hospital. All participants and their parents/legal guardians provided written informed assent and consent.

### 2.2. Clinical Assessment

All participants (*n* = 51) were assessed with the Schedule for Affective Disorders and Schizophrenia for School Aged Children Present and Lifetime Version (K-SADS-PL) [15], a semi-structured interview that assesses the presence of mental disorders. 

Subsequently all participants were assessed with the Children’s Yale–Brown Obsessive Compulsive Scale (CY-BOCS) [13], a clinician-rated semi-structured interview that assesses the presence and severity of obsessive–compulsive disorder. To categorized and critical analyzed obsessions and compulsions assessed through the CY-BOCS, we used the Four-Factor Model of Leckman [16]. The author organized the obsessions in four areas: Aggressive, Sexual, Religious, Somatic; Symmetry; Contamination and Hoarding. Also the compulsions were divided into four areas: Checking; Repeating rituals, Counting, Ordering, Arranging; Cleaning; Hoarding, Collection.

UHR symptoms were indexed on the Structured Interview for Psychosis-Risk Syndrome (SIPS/SOPS) [14]. The SIPS scales include a total of 19 symptom constructs (five positive, six negative, four disorganized, and four general symptoms) that are evaluated based on the presence, duration and severity of specific experiences and behaviors. Each of the five positive items is rated on a scale from 0 (absence of symptoms) to 6 (extreme or psychotic symptom intensity). A score of 3, 4, or 5 in at least one of the positive items indicates the presence of UHR symptoms. Scores of 0 in all five positive items indicated the absence of UHR symptoms.

The level of functioning was measured with the Childhood Global Assessment Scale (CGAS) [17]. Furthermore, social and role functioning was specifically assessed with the Global Functioning: Social Scale (GF: Social) [18] and the Global Functioning: Role Scale (GF: Role) [19] to obtain differential measures of functioning. The presence and level of depressive symptoms were measured using the Child Depression Inventory (CDI) [20]. 

Finally, neurocognitive functioning (IQ) was measured with the Wechsler Intelligence Scale for Children (WISC-IV) [21]. The Wisc-IV provides a measure of total intelligence (IQ) that can be analysed through four main indexes: Verbal Comprehension Index (VCI), Perceptual Reasoning Index (PRI), Working Memory Index (WMI) and Processing Speed Index (PSI).

### 2.3. Statistical Analysis

Data were analyzed using SPSS IBM Statistics version 20 statistical software (IBM Corp, Armonk, NY, USA). First, we calculated the prevalence of UHR symptoms in the sample (*n* = 51) of children and adolescents who met the criteria for OCD. Then, we divided the whole sample into two groups based on score on positive items of SIPS/SOPS: the first group was composed by children and adolescents with OCD that had a score of 3, 4 or 5 in at least one of positive items of the SIPS (Group OCD with UHR symptoms); the second group was composed by children and adolescent with OCD that had a score of 0 in all five positive items of the SIPS (Group OCD without UHR symptoms). 

The two groups were unequal in size, but Levene’s test confirmed homogeneity of variance and the Shapiro–Wilk test confirmed the normal distribution of the variables based on continuous data. Separate group comparisons based on one-way ANOVA were performed on demographic and psychiatric variables.

## 3. Results

### 3.1. Sample Characteristics

The sample consisted of 51 participants (mean age: 13.4 ± 2.9 years) with diagnosis of OCD. Although 56.9% of the sample did not report any UHR symptoms (Group OCD without UHR symptoms, 29 subjects), UHR symptoms were reported by 43.1% (Group OCD with UHR symptoms, 22 subjects) of the total sample. Of Group OCD with UHR symptoms, 45.4% reported suspiciousness/persecutory ideas, 72.7% unusual thought content/delusional ideas, 18.2% disorganized communication, 13.6% perceptual abnormalities, and 4.5% grandiose ideas. At the time of evaluation, no participant was subjected to pharmacological treatment.

### 3.2. Comparison between Groups

As shown in Table 1, there were no significant group differences for age (F [1,49] = 0.14, *p* = 0.700), education (F [1,49] = 0.87, *p* = 0.354), IQ (F [1,49] = 0.002, *p* = 0.96) or number of psychiatric diagnoses (F [1,49] = 0.459, *p* = 0.500). Also, there were no significant group differences for sex (χ^2^ = 0.30, *p* = 0.58). The two groups were also comparable for level of depressive symptoms (F [1,49] = 3.40, *p* = 0.072) and severity of OCD symptoms (both obsessions and compulsions) (F [1,49] = 0.52, *p* = 0.473).

Significant differences between the two groups were found in functioning. In fact, global functioning was worst in Group OCD with UHR symptoms (F [1,49] = 16.06, *p* = 0.0002); similarly, social and role functioning was lower in Group OCD with UHR symptoms (social functioning: F [1,49] = 9.4, *p* = 0.003762; role functioning: F [1,49] = 8.2, *p* = 0.006327).

In Table 2, we show the frequency and the percentage of obsessions and compulsions of our sample based on the Four-Factor Model of Leckman [16]. Both groups presented as the most prevalent type of obsessions the Aggressive, Sexual, Religious, Somatic type (50.0% group OCD with UHR symptoms; 58.6% group OCD without UHR symptoms) and as the most prevalent type of compulsions Repeating rituals, Counting, Ordering, Arranging type (45.5% group OCD with UHR symptoms; 48.3% group OCD without UHR symptoms). Statistical analysis confirmed no differences between group for obsessions (χ^2^ = 0.628; *p* = 0.87) and for compulsions (χ^2^ = 2.5587; *p* = 0.46).

In Table 3, we show the frequency and the percentage of non pharmacological treatment for both groups at the time of clinical assessment. Group OCD with UHR symptoms was characterized by a prevalence of patients that attended individual psychotherapy (68.2%), whereas the 41,4% of patients in the Group OCD without UHR symptoms not received any type of treatment and 34.5% attended individual psychotherapy. Statistical analysis confirmed no differences between group (χ^2^ = 7.69; *p* = 0.103).

## 4. Discussion

We found several clinically relevant results regarding the presence of UHR symptoms in a clinical sample of children and adolescents with OCD.

First, in our sample, the UHR symptoms are rather frequent; in fact, they appear in 43% of our children and adolescents with OCD. However, to the best of our knowledge, this is the first study aimed at investigating prevalence and clinical significance of UHR symptoms in a sample of children and adolescents with OCD. Further studies will be needed to confirm this data.

Indeed, our findings are not directly comparable with those of Niendam et al. [12]. This is due to differences between the two studies on the experimental design, tool instruments used for assessment and range-age of samples.

Children and adolescents with OCD and UHR symptoms also showed a more compromised global, social, and role functioning than children and adolescents with OCD but without UHR symptoms. This finding is all the more interesting if we consider that there were no significant differences between the two groups regarding age, IQ, the severity of obsessive symptomatology, the severity of compulsive symptoms, the number of psychiatric diagnoses (comorbidity) and the level of depressive symptoms. In addition, there were no significant differences between the two groups regarding psychosocial intervention type and all patients in both groups were dug-naïve patients at the time of clinical assessment. Therefore, the presence of UHR symptoms is associated with poorer global, social and role functioning independent of other clinical variables.

Although our results are initial data and other studies are required to replicate it, they raise the question of the need to evaluate the presence of UHR symptoms in patients with initial OCD diagnosis in order to plan therapeutic interventions to reduce functional impairment associated with their presence. In this context, it would be interesting to investigate any clinical differences on larger samples (e.g., through qualitative analyses of obsessions and compulsions and their relationship with contents of thoughts in UHR symptoms) and clinical outcome between children and adolescents with OCD and UHR symptoms and children and adolescents with OCD but without UHR symptoms.

The strengths of this study include the use of ‘gold standard’ instruments to assess psychiatric disorders. We used the K-SADS-PL [15], CY-BOCS [13], and SIPS/SOPS [14] that are semi-structured-interviews with levels of reliability and validity superior to those of self-report questionnaires. 

In particular, the administration of the SIPS/SOPS (positive items) [14] allowed us to investigate in depth the thought content of children and adolescents. Thus, we were able to distinguish qualitatively between possible aspects of magical thinking that are typical of OCD and UHR symptoms. 

This study also has several limitations: first, we did not have longitudinal data on the progression of UHR symptoms in our sample with OCD. Further study with larger sample sizes and follow-up data will be necessary to clarify the transition risk in OCD with UHR symptoms and how best to intervene. Second, we have no detailed information about the frequency of psychosocial interventions followed by patients in both groups. Further study must be performed for controlling this variable. Third, we did not investigate which clinical variables mediate the relationship between UHR symptoms and functional impairment. Despite these limitations, to the best of our knowledge, our study is the first to investigate the impact of UHR symptoms on global, social and role functioning in a wide age range of OCD patients (8–17 years old). 

## 5. Conclusions

Our findings suggest that the presence of UHR symptoms is an important element in the assessment and treatment of OCD because of their effect on social relationships, employment, and global functioning of patients. Indeed, first of all the clinician should recognize that OCD with UHR symptoms involves greater functional impairment than OCD not associated with UHR symptoms, and should then plan the appropriate non-pharmacological interventions. In fact, in our sample there are no differences in the way that the two conditions are treated. For example, children and adolescents with OCD and UHR symptoms could be supported with a cognitive-behavioral therapy (CBT) in order to improve the critical insight and awareness of cognitive biases about content of UHR symptoms. Indeed, as described in the Australian Clinical Guidelines for Early Psychosis [22], the focus of CBT in the UHR stage of psychosis enhances the understanding of symptoms (including, but not limited to psychotic symptoms) and strengthens resources for coping with strategies such as psychoeducation, challenging delusional thoughts and hallucinations with the aim of reducing the negative impact that these experiences have on the children and adolescents, stress management techniques, assertiveness or social skills training, and problem-solving skills.

## Figures and Tables

**Table 1:** Socio-demographic data and psychiatric assessment scores separately for two groups.

Variable	Group 1	Group 2	*p*-Value
	Patients OCD with UHR Symptoms	Patients OCD without UHR Symptoms	
	Mean (SD)	Mean (SD)	
Age, years	13.8 (2.6)	13.0 (3.2)	0.700
Education, years	8.4 (2.7)	7.6 (3.1)	0.354
IQ level	103 (18.6)	103 (11.9)	0.96
Number of psychiatric diagnoses	1 (0.6)	1 (0.9)	0.500
Total CY-BOCS score	23.6 (8.4)	25.5 (8.0)	0.473
SIPS Positive score	7.8 (2.9)	1.8 (1.4)	0.00001^1^
Total CDI score	17.0 (10.5)	11.9 (7.6)	0.072
C-GAS	45.9 (3.1)	53.7 (8.5)	0.0002^1^
GF: Social	3.7 (0.4)	4.3 (0.8)	0.003762^1^
GF: Role	3.8 (0.4)	4.3 (0.8)	0.006327^1^

*p* ≤ 0.0001. C-GAS, Children’s Global Assessment Scale, GF: Social, Global Functioning: Social Scale; GF: Role, Global Functioning: Role Scale; CDI, Child Depression Inventory; CY-BOCS, Children’s Yale–Brown Obsessive–compulsive Scale; SIPS, Structured Interview Prodromal Syndromes; UHR, Ultra High Risk; OCD, obsessive–compulsive disorder

**Table 2:** Frequency and percentage of obsessions and compulsions based on Four-Factor Model [16].

	Group OCD with UHR Symptoms (*n* = 22) *n* (%)		Group OCD without UHR Symptoms (*n* = 29) *n* (%)	
**Obsessions**				
Aggressive, Sexual, Religious, Somatic	11	(50.0%)	17	(58.6%)
Symmetry	9	(40.9%)	9	(31.0%)
Contamination	8	(36.4%)	12	(41.4%)
Hoarding	1	(4.5%)	2	(6.9%)
**Compulsions**				
Checking	9	(40.9%)	12	(41.4%)
Repeating rituals, Counting, Ordering, Arranging	10	(45.5%)	14	(48.3%)
Cleaning	8	(36.4%)	13	(44.8%)
Hoarding, Collection	1	(4.5%)	7	(24.1%)

**Table 3:** Frequency and percentage of non pharmacological treatment scores separately for two groups.

	Group OCD with UHR Symptoms (*n* = 22) *n* (%)		Group OCD without UHR Symptoms (*n* = 29) *n* (%)	
Individual psychotherapy	15	(68.2%)	10	(34.5%)
Familiar therapy	0	(0%)	1	(3.4%)
Individual psychotherapy and Familiar therapy	3	(13.6%)	4	(13.8%)
Individual psychotherapy and Parent training	2	(9.1%)	2	(6.9%)
None	2	(9.1%)	12	(41.4%)
